# Supplementary material for: Specific origin selection and excess functional MCM2–7 loading in ORC-deficient cells
Source: Nucleic Acids Res. 2025 Jun 18;53(11):gkaf518. doi: 10.1093/nar/gkaf518 (PMC12203792; doi:10.1093/nar/gkaf518)

Supplementary figure S1 Permutation test results of colocalization between various origin peaks and simple repeat regions with TACC motif.

Supplementary figure S2 ORC2 WB in ORC2 KO cells. A**)** Immunoblot of ORC2 in ORC3 IP in ORC2KO cell line. B**)** Immunoblot of ORC2 in ORC2 KO cells with different ORC2 antibodies

Supplementary Table S1 Adjusted p-values and 95% confident intervals of Welch's t-tests (**Fig. 5A, 6 and 7**).

Supplementary Table S2 Adjusted p-values and effect sizes of comparison tests (**Fig. 4**). Bold: Effect size > 0.15 or <-0.15

Supplementary Table S3 Adjusted p-values of the Kolmogorov-Smirnov test, and Wasserstein distance (**Fig. 5B-D**).

Supplementary Table S4 MCM2 Fluorescence signals in G1-MCM^{DNA} positive cells used for Ergodic Rate Analysis calculation. Each value is fluorescence intensity for MCM2 in one cell. There are 3 biological replicates for each cell line.

Supplementary Fig. S1

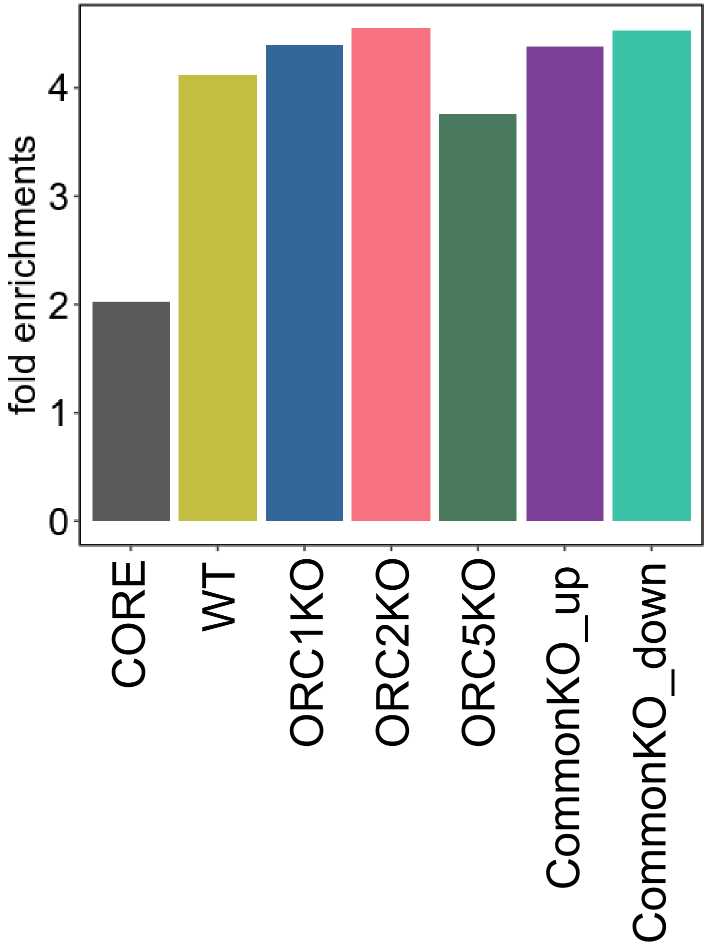

Supplementary Fig. S2

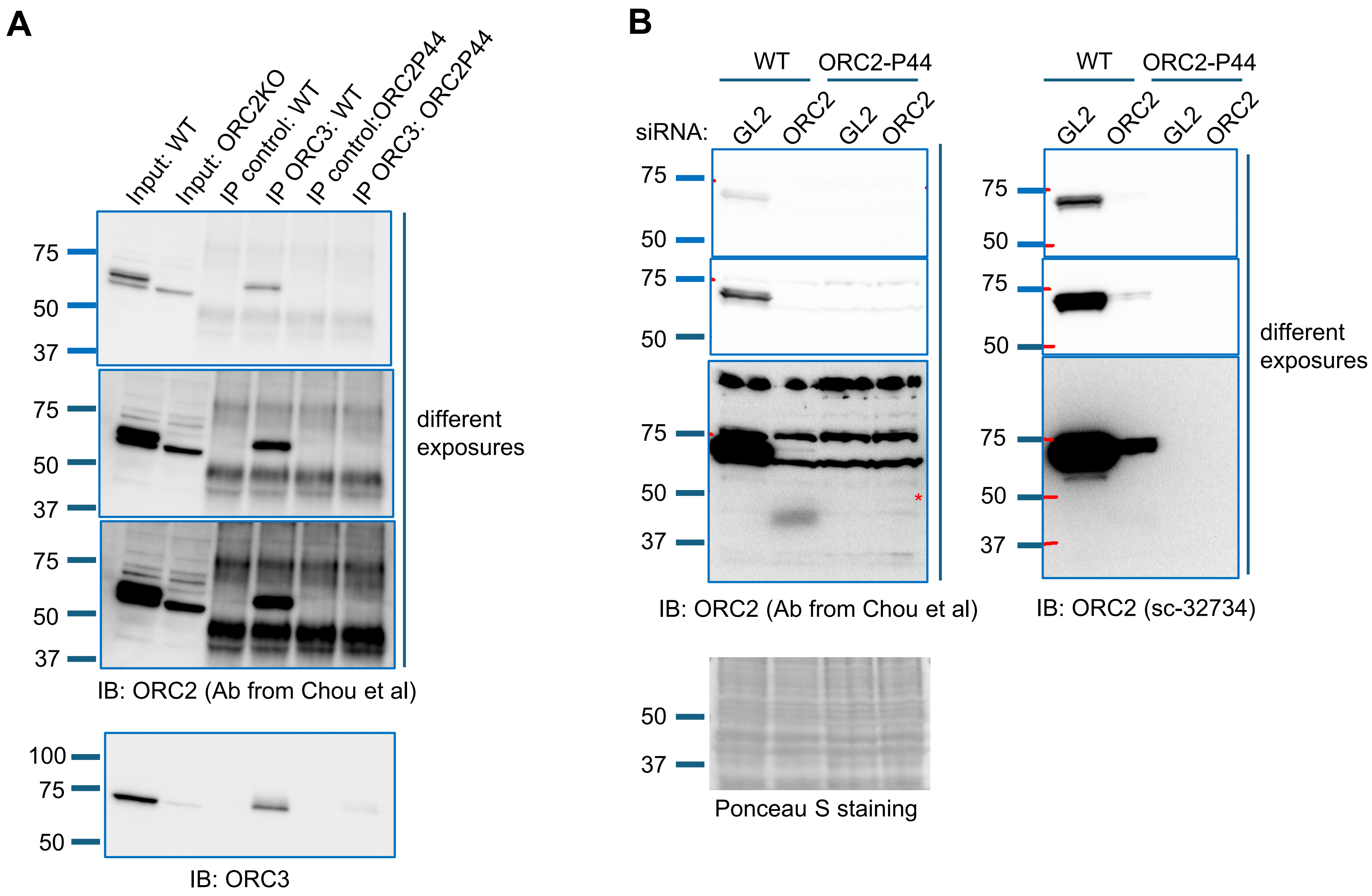

Supplement: gkaf518_Supplemental_Files [file gkaf518_supplemental_files.zip › supplementary figures.pdf]
